# Executive Function in Fragile X Syndrome: A Systematic Review

**DOI:** 10.3390/brainsci9010015

**Published:** 2019-01-16

**Authors:** Lauren M. Schmitt, Rebecca C. Shaffer, David Hessl, Craig Erickson

**Affiliations:** 1 Division of Child and Adolescent Psychiatry, Cincinnati Children’s Hospital Medical Center, Cincinnati, OH 45229, USA; lauren.schmitt@cchmc.org; 2 Division of Developmental and Behavioral Pediatrics, Cincinnati Children’s Hospital Medical Center, Cincinnati, OH 45229, USA; rebecca.shaffer@cchmc.org; 3 Department of Pediatrics, University of Cincinnati College of Medicine, Cincinnati, OH 45267, USA; 4 MIND Institute, Department of Psychiatry and Behavioral Sciences, University of California, Davis, CA 95616, USA; drhessl@ucdavis.edu; 5 Department of Psychiatry and Behavioral Neuroscience, University of Cincinnati College of Medicine, Cincinnati, OH 45267, USA

**Keywords:** fragile X syndrome, executive function, working memory, set-shifting, cognitive flexibility, inhibitory control, attention, planning, processing speed

## Abstract

Executive function (EF) supports goal-directed behavior and includes key aspects such as working memory, inhibitory control, cognitive flexibility, attention, processing speed, and planning. Fragile X syndrome (FXS) is the leading inherited monogenic cause of intellectual disability and is phenotypically characterized by EF deficits beyond what is expected given general cognitive impairments. Yet, a systematic review of behavioral studies using performance-based measures is needed to provide a summary of EF deficits across domains in males and females with FXS, discuss clinical and biological correlates of these EF deficits, identify critical limitations in available research, and offer suggestions for future studies in this area. Ultimately, this review aims to advance our understanding of the underlying pathophysiological mechanisms contributing to EF in FXS and to inform the development of outcome measures of EF and identification of new treatment targets in FXS.

## 1. Introduction

Fragile X syndrome (FXS) is the leading inherited monogenic cause of intellectual disability, resulting from >200 CGG trinucleotide repeat expansions in the 5′ untranslated region of the Fragile X Mental Retardation 1 Gene (*FMR1*). The resulting hyper-methylation and silencing of FMR protein (FMRP) production disrupts synaptic structure and function [1,2,3,4,5], leading to neural hyper-excitability and atypical brain development. The characteristic phenotypic features in humans with FXS, including impaired cognition, are hypothesized to be downstream effects of the altered neurodevelopment [6,7]. Because FXS is an X-linked genetic disorder, females with FXS are typically less severely affected than males with FXS due to the variability of X-chromosome inactivation in females [8]. Thus, females with FXS who demonstrate a greater degree of methylation and lower FMRP levels have a phenotype more similar to males with FXS, whereas females with less methylation and greater FMRP levels demonstrate more subtle clinical features. This is consistent with numerous documentations that the severity of cognitive impairments are associated with the degree of methylation mosaicism and FMRP expression in individuals with FXS [9,10,11,12], suggesting a progressive FMRP deficit leading to cognitive impairments. Yet, the precise mechanisms underlying specific cognitive impairments remain poorly understood in FXS.

Executive function (EF), or a group of discrete cognitive abilities that support adaptive goal-directed behavior [13], has been consistently documented as impaired in individuals with FXS, even beyond what is expected given their general cognitive impairments (for examples, see [14,15,16,17,18,19,20,21,22,23]). Whether this reflects a generalized deficit in EF or multiple deficits to specific EF domains (i.e., working memory, response inhibition, cognitive flexibility, attention, planning, and processing speed) is less clear. Previous studies using parent-report questionnaires have consistently documented high rates of inattention and hyperactivity in FXS, but these studies have seldom used questionnaires targeting a broader range of EF impairments or other questionnaires to assess additional EF domains (for examples, see [24,25]). In contrast, previous studies using traditional neuropsychological and experimental performance-based measures of EF have been able to capture deficits across all domains of EF in individuals with FXS. Behavioral performance-based measures have a distinct advantage over parent-report measures in their potential to be translated into tasks used during brain imaging studies (i.e., functional magnetic resonance imaging (fMRI) or electroencephalogram (EEG)) or into analogous versions to be used in studies of rodent models of FXS (i.e., *FMR1* KO mouse). Further, compared to parent-reports, performance-based measures are better able to objectively quantify performance, which may help provide insights into the specific brain regions affected in FXS as well as identify EF deficits that may be specific to FXS rather than those related to general cognitive impairments and/or common comorbid diagnoses, like attention deficit/hyperactivity disorder (ADHD) and autism spectrum disorder (ASD). Given previous indications of abnormalities in the frontostriatal regions supporting EF in FXS (for example, see [26]), translational studies of EF offer great promise for use as quantifiable biomarkers of brain function useful for early-phase drug development and intervention trials.

Despite over 50 studies examining EF deficits in FXS using performance-based measures, a comprehensive review of findings from males and females with FXS and their implications still is needed (Table 1). Generally, previous studies of EF in FXS documented impairments across each domain of EF relative to both chronologically age-matched (CA) controls and mentally aged-matched (MA) controls, yet closer examination reveals key differences in performances depending on domain, measure, task difficulty, sex, and/or control group studied. Understanding specific profiles of executive dysfunction in FXS whose etiology may be distinct, yet overlapping, with that of general cognitive impairments, is critical to understanding pathophysiological processes in FXS and developing novel treatments for this patient population. Though EF impairments are major cause of distress for individuals with FXS and their families [27,28] and poor EF leads to worse learning and academic achievement outcomes [29,30], the development of behavioral and pharmacological interventions aimed at improving EF have lagged behind those targeted towards other key phenotypes, like anxiety and sensory hyper-sensitivity. Thus, a comprehensive review of previous studies is needed to summarize EF deficits and their clinical and biological correlates in FXS, establish potential underlying brain mechanisms of these deficits, and address critical limitations of previous studies. As more clinical research begins to use EF as outcome measures in treatment trials, it is crucial to review previous studies to guide future research studies examining EF in individuals with FXS. 

## 2. Methods

### 2.1. Search Strategy

To identify studies for inclusion in the review, computerized databases, including PubMed and PsychINFO, were used to conduct searches. Keywords such as “Fragile X” AND “executive function”, “working memory”, “inhibitory control”, “cognitive flexibility”, “set-shifting”, “attention”, “processing speed”, or “planning” as well as variants on these terms were used. After collecting all available peer-reviewed published articles, their reference sections were scanned to identify additional articles that may have been previously missed. Authors of articles that were not available were contacted.

### 2.2. Selection Criteria

Broad inclusion criteria were used in order to provide the most comprehensive review of the literature. The following criteria were used to determine whether an article could be included in the review:

1. The paper was published in a peer-reviewed journal,
2. At least one participant sample had full mutation FXS,
3. Performance of individuals with FXS was compared against either a control population or normative sample data OR performance of individuals with FXS is documented in the context of a feasibility study,
4. At least one measure of EF was used,
5. The study reported quantitative scores (e.g., raw score, T-score, Standard score, etc.) beyond completion rates,
6. EF was a primary or secondary research question, and
7. The study was not a case study.

### 2.3. Study Organization and Consolidation

The studies and corresponding measures were organized into six executive domains commonly reported in the literature: working memory, response inhibition, cognitive flexibility, attention, processing speed, and planning. In the first section, we summarized primary findings within each executive function domain, separated by males and females (Table 1) and then in relation to clinical and psychological variables. Next, we discussed current knowledge regarding neurobiology of EF deficits in FXS by reviewing biological correlates, findings from studies using brain imaging and rodent models, and potential pathophysiological mechanisms underlying these deficits. Finally, we addressed critical considerations from previous studies and provide recommendations for future studies. 

## 3. Review

### 3.1. Executve Function Deficits

#### 3.1.1. Working Memory

Working memory is necessary for temporarily storing information for immediate use, like remembering the homework assignment the teacher announced, and it often requires the manipulation of that information, such as remembering to bring home the textbook needed for the assignment. Theories propose a phonological loop aids in storage of verbal information, while a visuospatial sketchpad aids in storage of visual-spatial or visual-perceptual information [80]. Here, we discuss working memory measures based on the type of information needed to be retained: verbal or nonverbal (i.e., spatial and perceptual). 

Verbal working memory was the most common EF domain assessed across FXS studies, with the majority of studies reporting impairments in males with FXS from school-age to adulthood compared to both chronological age (CA) and mental age (MA) control groups [15,17,22,31,32,33,34,35,36,37,38,39,40,41]. Among males with FXS, the type of stimuli (i.e., words, digits) was less influential on performance than task difficulty, or cognitive load, and this pattern emerged regardless of age. Cognitive load can depend on the amount of information needing retention or the degree of manipulation of information needed. For example, verbal working memory was relatively intact when cognitive load was low, like remembering two to five words in forward order. In contrast, verbal working memory was significantly impaired in males with FXS when cognitive load was high, like remembering the first word from two different five-word lists [21,41,42]. This finding is consistent with studies documenting males with FXS had a greater likelihood of hitting floor effects on higher load verbal working memory tasks [22,31,32]. Similar findings also arose in some studies comparing males with FXS to other syndromic participant groups (e.g., individuals with Down syndrome (DS)). For example, males with FXS performed similar to males with DS when working memory load was either very low (e.g., digit span forward; [17,41] or very high (e.g., story retelling; [17]). This suggests both groups had relatively intact abilities when working memory demands were low, whereas both groups were equally taxed compared to CA controls under high working memory demand conditions. Yet, under moderate verbal working memory conditions, males with FXS performed worse compared to males with DS, suggesting verbal working memory deteriorated more rapidly with task difficulty in FXS than in DS. However, other studies do not support this finding, and instead suggest task difficulty may have been less influential within syndromic groups [15,35,47,48], warranting future studies that systematically vary difficulty to clarify these inconsistencies. 

With regards to development, two studies reported verbal working memory developed slower in young males with FXS compared to CA controls even after accounting for mental age [36,50]. However, in contrast, another study documented a narrowing gap in verbal working memory performance in male and female children with FXS compared to a normative sample [33]. These contradictory findings may be accounted, in part, by the presence of females in the latter sample. Alternatively, because the narrowing gap was documented in an older sample of children (up to age 16 years), it also is possible that verbal working memory performance in males with FXS developed more slowly during early and middle childhood, then improved dramatically during adolescence. Visual inspection of growth curves demonstrating a relatively flat maturation rate from 6–12 years, followed by a rapid increase in performance beginning around 12 years confirmed this assertion [33]. Still prior studies have documented impaired verbal working memory in adult males with FXS, suggesting that though performance may become more similar to CA controls, it is nonetheless impaired. Overall, previous studies of verbal working memory in males with FXS suggest performance is highly dependent on cognitive load with more severe impairments as the amount of information is increased or when manipulation is required and, to some extent, chronological age. However, verbal working memory deficits may not be specific to FXS relative to other syndromic disorders.

With regards to nonverbal working memory, both visual–spatial (i.e., remembering the location of an item) and visual–perceptual (i.e., remembering what item was previously shown) subdomains are impaired in males with FXS relative to both CA and MA control groups [16,21,22,31,32,42,48]. As with verbal working memory, performance depended on cognitive load, with relatively intact abilities when demands were low, like remembering two locations in an array, but significantly worse abilities compared to controls when demands were high, like remembering three locations on two different arrays [21,42,49,51]. Notably, one study reported a relative strength in visual–perceptual compared to visual–spatial working memory in males with FXS [48], suggesting the ability to remember what object was shown is stronger than the ability to remember where the object was located. Young males with FXS demonstrated slower development of nonverbal working memory compared to CA controls after accounting for mental age [36,50], suggesting deficient growth of skills. Still longitudinal studies of nonverbal working memory have not yet been completed in older children and adult males with FXS, suggesting performance may improve more rapidly in later develop as seen in verbal working memory. Most studies found that males with FXS performed similarly to other syndromic participant groups on measures of nonverbal working memory, [15,35,47,48] with some evidence of worse performance than syndromic control groups during moderate level tasks [17,41] as found in studies of verbal working memory. Taken together, this suggests verbal and nonverbal working memory deficits are present in males with FXS across the lifespan that worsen with increased cognitive load, but are unlikely specific to this syndromic population.

Few studies examined verbal and nonverbal working memory in females with FXS. Verbal working memory performance was found to be impaired relative to CA controls [33,38] but relatively intact compared to age- and IQ-matched [43] as well as environmental (i.e., unaffected family member) control groups [34,39,40,44]. Though neither stimulus type nor cognitive load emerged as relevant factors in performance. One study documented verbal working memory performance became more similar to a normative sample from school age to late adolescence in females with FXS; however, since males also were included in the sample, it is difficult to determine whether these developmental findings occurred in one or both sexes [33]. Visual–spatial working memory abilities in females with FXS also were impaired compared to CA controls, and unlike verbal working memory abilities, seemed to depend on cognitive load as found in males with FXS [49,51]. For example, females with FXS performed worse than CA controls when required to match the spatial position with that of two trials ago (i.e., two-back) but similarly to controls when required to match with one trial ago (i.e., one-back) [49]. With regards to visual-perceptual working memory, studies reported females with FXS showed impairments relative to age- and IQ matched [43] and environmental control groups [34]. However, intact visual-perceptual working memory also has been documented compared to an environmental control group [44]. With fewer studies completed in FXS females, inconsistent findings make interpretations regarding verbal and nonverbal working memory less clear. Still previous research suggests working memory performance may be more variable in females with FXS consistent with their wide spectrum of general cognitive and adaptive abilities.

When comparing verbal and nonverbal working memory, some [32,36,42] but not all [41,51] studies found verbal working memory to be a relative weakness compared to nonverbal working memory in males with FXS, whereas the opposite pattern emerged in females with FXS [34,43,51]. For example, Pierpont and colleagues documented females with FXS outperformed males with FXS on verbal working memory measures regardless of cognitive load [45]. The finding supports studies documenting stronger expressive language skills in females with FXS compared to males with FXS [81] as well as more consistent findings of nonverbal, as opposed to verbal, working memory impairments in females with FXS. Though future studies are warranted to confirm these observations, as these findings may have distinct treatment implications for males and females with FXS in terms of areas targeted and strategies used. 

#### 3.1.2. Inhibitory Control 

Inhibitory control refers to the ability to suppress contextually-inappropriate responses, and it is critical for adapting behavior to changing and often uncertain environmental demands. Two distinct cognitive components comprise inhibitory control: prepotent response inhibition (i.e., the ability to withhold a previously reinforced behavior) and distractor interference (i.e., the ability to ignore irrelevant information) [82], each of which have been evaluated in FXS.

Studies consistently documented reduced ability to withhold prepotent behavioral responses in young male children with FXS compared to both MA and CA controls [14,16,52,53]. Fewer studies have been conducted with older children and adolescent males with FXS, and findings have been more inconsistent, demonstrating both intact [54] or impaired performance [55]. However, intact performance only was reported relative to an age- and IQ-matched DD control group during an fMRI task, suggesting cognitive and behavioral issues may have been less severe in this sample of males with FXS since they were able to complete an fMRI session [54]. Thus, it is more probable that prepotent inhibition deficits persists into adolescence in males with FXS, as supported by findings from longitudinal and cross-sectional studies [14,52,55]. Prepotent inhibition errors also occurred at higher rates among males with FXS relative to both males with William Syndrome (WS) and males with DS during school-age and adolescence [16,52]. This suggests withholding prepotent behavioral responses may be more severely affected in FXS relative to other syndromic groups. Though whether this reflects different developmental trajectories and/or persists into adulthood is unclear. 

Impairments in distractor interference were documented from preschool-age to adulthood in individuals with males with FXS compared to CA-, MA-, and IQ-matched control groups [16,22,31,35,52,57] (but see [55] for null findings). This suggests difficulties inhibiting behavioral responses to environmental distractors are present early in childhood and persist into adulthood in males with FXS regardless of measure used (e.g., Stroop, Flanker). Findings that distractor interference improved a rate similar to MA controls during early childhood suggest attenuated maturation of underlying brain processes emerge early in development, but whether similar findings are observed during later childhood to adulthood remains unclear. Comparisons with other syndromic populations suggested distractor interference was relatively similar in males with FXS and males with WS [52] but impaired compared to males with DS [16,35]. Though, the study of males with WS was completed in preschool-age participants, whereas the studies of males with DS included school-age to adult participants, suggesting inconsistencies may have emerged regarding differences in ages studied or actual differences in the developmental trajectories of distractor interference. Taken together, since males with FXS demonstrated impaired prepotent response inhibition and distractor interference regardless of measure used (e.g., antisaccade, go/no-go), this suggests failure to inhibit behavioral responses may be less dependent on task stimuli and complexity, thus making measurement selection in future studies less constrained.

Only one study of prepotent response inhibition has been completed in females with FXS, in which behavioral performance during an fMRI task was similar to that of female CA controls [26]. This suggests inhibiting prepotent behavioral responses may be relatively intact in females with FXS, though participant recruitment may have been biased towards less affected females due to using an fMRI protocol. Studies assessing distractor interference in females with FXS also were equivocal, with some findings of higher error rates during Stroop-like measures compared to environmental controls and IQ-matched controls [34,57,58] and others of similar error rates to CA controls and IQ-matched controls [59,60]. This suggests inhibiting responses towards distractors may be more variable in females with FXS, consistent with their wider range of functioning and general cognitive impairments. Of note, Tamm and colleagues’ observed females with FXS to have a similar distractor interference error rate to CA controls, but they were slower to respond [60], suggesting some females with FXS may have adopted a strategy to sacrifice speed for sake of accuracy. This cognitive strategy to slow responses in order to improve poor inhibitory control was not observed in males with FXS, suggesting this compensatory strategy may only be present in less affected individuals, like females with FXS. Still, future studies are warranted to better understand potential compensatory strategies to improve inhibitory control in males and females with FXS.

Though several studies examined both prepotent response inhibition and distractor interference performance [16,52,55], no study to date has directly compared these abilities directly in individuals with FXS. Thus, whether prepotent response inhibition or distractor interference is relatively more impaired than the other or whether they both reflect a more general impaired process of behavioral inhibition is not yet clear. Though Woodcock and colleagues documented impaired prepotent response inhibition in school-aged/adolescent males with FXS relative to CA controls, but intact distractor interference [55], it is important to note that authors used different variables for each measure. For example, error rate was used to assess performance on the prepotent response inhibition measure and reaction time was used to assess performance on the distractor interference measure. Thus, comparisons between domains are less appropriate since they are quantifying difference aspects of inhibitory processes. Furthermore, to our knowledge, no study to date has compared performance between males and females with FXS. Hessl and colleagues [35] reported impaired performance on a distractor interference measure in males and females with FXS relative to CA, DS, and iDD controls; however, results were not provided for each sex. Thus, clarifying patterns of deficits between prepotent response inhibition and distractor interference as well as between sexes is needed and will be important for underlying the discrete brain mechanisms disrupted in FXS as well as informing potential treatment targets.

#### 3.1.3. Cognitive Flexibility 

Cognitive flexibility refers to the ability to adaptively change behavior based on contextual demands. This is most commonly assessed with dimensional sorting tasks in which participants are required to switch from sorting figures based on one intra-dimensional feature (e.g., spatial location on screen) or extra-dimensional feature (e.g., color or size) to another. Difficulty switching is most commonly quantified by calculating the total number of trials to reach a specified criterion (i.e., higher number indicates worse performance) or by number of categories achieved (i.e., lower number indicates worse performance). However, several studies also calculated error rates, with a few categorizing errors as either perseverative (i.e., continuing to choose previously correct responses despite feedback that it is no longer correct), regressive (i.e., returning to the previously correct response after establishing the new correct response), or distractor (i.e., choosing a never-reinforced or distractor response). 

Across studies, males with FXS performed worse on cognitive flexibility measures compared to CA and MA controls [22,31,32,35,48,55,83]. Deficits were observed from school-age to adulthood, though error rates reduced with increasing MA in males with FXS [31]. Several studies also found that impaired performance was predominantly due to increased perseveration errors [34,48,83]. This suggests individuals with FXS have difficulty shifting away from previously rewarded responses and choosing new responses even after the previous response is no longer rewarded. This finding from cognitive performance-based measures is consistent with findings from clinical-ratings that report increased perseverate speech and behavior in individuals with FXS. Interestingly, Van der Molen and colleagues [46] reported that perseverative responses were more prominent when cognitive demands were low, whereas distractor errors were more prominent as cognitive demands increased. This provides additional evidence that males with FXS have a perseverative response style, which may reflect failure to disengage attention from a previously reinforced stimulus even when it is no longer rewarded. Yet, when too many distractors were present, impaired distractor interference (an aspect of inhibitory control) contributed to inflexibility, and thus attention was more readily diverted towards irrelevant stimuli. This importantly demonstrates certain EF measures may require more than one type of EF, and that individuals with FXS may be more disadvantaged during these certain measures since multiple EF domains are disrupted.

Of note, Garner and colleagues reported absence of cognitive flexibility deficits in school-age males with FXS relative to age- and IQ-matched controls during a modified version of the Wisconsin Cart Sorting Task (WCST-M) created for individuals with cognitive impairments [61]. This suggests cognitive flexibility may be intact relative to individuals of similar general cognitive impairments. It also suggests the adapted versions of measures may be more appropriate for FXS participants, and may reflect a better estimate of true cognitive flexibility skills as it requires less recruitment of other EF domains. Inconsistent findings emerged comparing cognitive flexibility performance in males with FXS relative to other syndromic groups, with some studies documenting more impaired performance in males with FXS compared to males with idiopathic ID and males with DS [15] and others documenting intact performance compared to these groups as well as males with Prader–Willi Syndrome (PWS) [35,48,55]. Though these findings suggest deficits in cognitive flexibility may not be specific to FXS, Van der Molen and colleagues conducted a discriminant analysis to classify error types during a dimensional sorting task to differentiate groups and found that perseverative responses differentiate males with FXS from DS and MA control groups [46]. Thus, though cognitive flexibility impairments may not be specific to males with FXS, their perseverative response style may be unique to this patient population, implicating a critical area to be further explored in future studies.

Few studies of cognitive flexibility have been conducted with females with FXS. Still, cognitive flexibility deficits were documented in females with FXS relative to environmental controls [23,34], and like their male counterparts, these deficits predominantly emerged due to increased rate of perseverative errors in one [34] but not the other study [23]. Thus, it is possible that a subgroup of females with FXS may show cognitive flexibility deficits similar to those in males with FXS, though more studies are needed. 

#### 3.1.4. Attention

Attention refers the ability to concentrate awareness with a specific behavioral or cognitive goal. For example, sustained attention is needed to maintain general focus, selective attention is required when concentrating on a specific information while ignoring other, and divided attention is used when focus is required towards multiple goals. Across studies, a variety of measures were used and quantified attention performance in different ways. The most common variables were: reaction time (time to respond to target), hit rates (correct target identification), miss rates (omission of target identification), and false alarm rates (incorrect target identification). Though most studies examined visual attention towards visual stimuli, a few also examined auditory attention.

In school-aged males with FXS, sustained attention was found to be impaired relative to CA and MA controls as they demonstrated both slower reaction times [16,50,52,62] and reduced hit rates [50,62]. One study reported reduced reaction time in the absence of differences in hit rate [16], suggesting some males with FXS may attempt to slow behavioral responses in order to perform more accurately. Still this cognitive strategy to improve accuracy was not observed in other studies, suggesting the majority of males with FXS may not slow responses at all or enough to be effective [50,62]. Sustained attention was impaired across both the auditory and visual domains, though males with FXS demonstrated relatively weaker performance (fewer correct hits and fewer correct rejections) during auditory compared to visual measures [62,63], consistent with findings of strong visual-perceptual skills and disturbances in auditory processing in males with FXS [84]. School-age males with FXS showed comparable performance to MA controls in one study that used a shorter version of a sustained attention measure [63], suggesting males with FXS may be able to maintain attention for a specific behavioral goal for a short duration (<5 min), but have greater difficulty when tasks require longer durations of sustained attention. Additionally, relatively intact sustained attention also was reported in males with FXS from 11–38 years, suggesting sustained attention may be less impaired in later childhood into adulthood [37]. Longitudinal studies in young males with FXS reported sustained attention developed at a slower rate compared to CA controls, but at a similar rate when adjusted for MA [50]. Yet, whether gaps in performance relative to CA controls narrows with age remains unclear. Previous studies reported sustained attention was similar among males with FXS and males with DS [16,52], but stronger in males with FXS compared to males with WS [52] in terms of both hit rates and reaction times. Taken together, previous studies have indicated consistent findings of poor sustained attention among school-age males with FXS though performance may be less impaired when tasks are shorter and with increasing age.

During selective attention tasks, previous studies have reported similar hit rates in males with FXS relative to MA controls from toddlerhood to adulthood [15,18,50,52,65]. However, a few studies documented worse performance compared to MA [16,19,65] and CA controls [19], especially under certain conditions. For example, males with FXS had lower hit rates and increased errors when more distractors were present and/or when distractors were more similar to target stimuli. A similar finding was documented during studies of distractor interference, suggesting both selective attention and inhibitory controls skills may be highly dependent on context of the environment. For example, certain EF abilities are relatively intact in a less distracting and ambiguous setting for males with FXS, but as the cognitive and neural processes supporting these abilities become more taxed, they are more likely to make errors. Of note, multiple studies reported that males with FXS demonstrated increased rates of perseverative responding during selective attention measures, or responding to the same correct target multiple times even after the end of the trial [52,65]. This finding is consistent with studies of cognitive flexibility and distractor interference that also reported a higher number of perseveration errors relative to all control groups. This suggests males with FXS demonstrated both impaired ability to shift behavior and attention away from previously correct responses even in the absence of reinforcement. Taken together, these findings indicate that a similar underlying cognitive and neural mechanism may drive perseverative responding and/or failures to disengage. Further evidence that this response style is specific to males with FXS comes from selective attention studies demonstrating increased perseverative errors compared to both DS [18,52] and WS groups [19,52] even when overall performance was similar across groups [18,19]. Yet, not all previous selective attention studies categorized error types, suggesting additional studies are needed to confirm these findings [16,19]. Though one study reported stronger selective attention performance in adult males with FXS compared to adult males with DS [83], it is unclear whether this reflects greater improvements in selective attention from adolescence to adulthood relative to individuals with DS or differences in measures used [18]. Overall, previous studies noted relatively intact selective attention in males with FXS across the lifespan that becomes impaired as task difficulty increases. Yet, the presence of perseverative errors suggests a more subtle impairment in this area that may be related to other areas of executive dysfunction as well as specific to FXS.

Only one study comparing divided attention performance to MA controls found fewer hits but similar response times and rates of false alarms [16]. Authors also documented greater distance moved when locating stimuli [16], which may account, in part, for the lower hit rate. More studies are warranted in assessing divided attention in FXS. Studies examining attention in females with FXS are needed to determine whether their profile of deficits is similar to those in males with FXS.

#### 3.1.5. Planning

Planning is the ability to manage current and future goals and involves formulation, selection, and execution of specific sets to reach those goals. Few studies have examined planning in individuals with FXS, which may be, in part, due to prominent floor effects [32]. This suggests available planning measures may be too difficult for individuals with FXS to complete and/or planning abilities are significantly more impaired in FXS relative to other domains of EF. During a version of the Tower of Hanoi task, school-age males with FXS were observed to achieve fewer correct trials compared to MA controls, suggesting males with FXS had an impaired ability to plan or problem solve during increasingly complex scenarios [22,31]. Still, future studies are warranted in this area, especially those that include females with FXS, and studies utilizing tests with lower floors to measure performance of a broader range of individuals. Additionally, assessment of error types may be useful in future studies as has been found in measures of other EF domains.

#### 3.1.6. Processing Speed

There is some controversy whether processing speed is an executive function or a cognitive process that supports all executive functions (for example, see [85]). For the purpose of this review and its relevancy in FXS, we include processing speed as a component of EF, using the more conservative definition of simple reaction time [85]. Processing speed performance varied across studies but was highly dependent on control group used. For example, males with FXS from school-age to adulthood demonstrated longer reaction times compared to CA controls [33,35], but comparable reaction times to MA, iDD, and DS control groups [22,31,35]. This is consistent with findings that reaction times increased at a slower rate relative to CA [33] but at a similar rate relative to MA controls in FXS males [31]. Longer reaction times also were observed for females with FXS relative to CA controls, but it is unclear whether performance differs from MA controls [33,35]. Together, this suggests processing speed is deficient compared to typical development, but impairments are likely not specific to FXS.

#### 3.1.7. Behavioral and Psychological Correlates

Parent-report measures used to identify clinical relationships include those that exclusively examined EF dysfunction (e.g., Behavior Rating Inventory of Executive (BRIEF)), those that contained subdomains related to EF impairment (e.g., hyperactivity subscale of the Aberrant Behavior Checklist (ABC)), and those examining other aspects of behavior and function, like daily living skills. Most studies demonstrated significant relationships between behavioral performance and parent-report measures, though associations were not limited to the specific EF domain behaviorally assessed [14,35,56]. For example, impaired prepotent response inhibition was associated with parent-reports of inattention and hyperactivity and impaired distractor interference was associated with parent-reports of inattention, hyperactivity, stereotyped speech, and reduced adaptability to change [14,56]. Likewise, cognitive flexibility related to attention problems and adaptability to change [56] and processing speed related to hyperactivity, stereotyped speech, and reduced adaptability to change [56]. This extensive overlap in parent-reported clinical presentation and behavioral performance has several implications. First, it suggests that the EF domains captured in parent-report measures may not correspond well to those domains examined using behavioral measures. For example, difficulty flexibly shifting away from a previous behavioral response during a cognitive flexibility task may be reported by parents as difficulty shifting attention. This also could mean that both or either lack discriminant validity, reflecting measurement specificity issues. Second, the extensive overlap in symptoms may reflect less differentiated brain processes underlying separate EF domains in FXS typically found in younger typically developing children. It also may reflect multiple neural system dysfunctions. Lastly, it also could suggest that behavioral measures used in studies are likely confounded by co-occurring conditions, like ADHD, ASD, or anxiety. For instance, difficulties in sustained attention and cognitive flexibility may interfere with performance during a Stroop-like task. Similarly, studies examining EF performance in relation to ASD symptom severity provided consistent findings of more severe ASD symptomalogy relating to worse EF deficits, which was relatively independent of domain [31,35,36,63,64]; (but see [38] for null finding). Of note, Cornish and colleagues reported that poorer auditory, but not visual, attention predicted more severe ASD symptoms 12 months later, suggesting auditory attention may be an important risk marker for ASD in young males with FXS [64]. Future studies are needed to clarify these relationships by directly comparing FXS participants with and without co-occurring diagnoses. Additionally, since these studies examined correlations without controlling for MA or global IQ, it also will be important for future studies to determine the extent to which some of these relationships are driven by more general cognitive deficits than those related specifically to EF.

Additionally, adaptive behavior skills were related to inhibitory control as well as verbal and nonverbal working memory in a co-ed FXS sample [35] and to inhibitory control in a female only sample [59]. For example, working memory deficits could make performing self-care routines challenging without visual reminders of necessary steps to bathing or brushing teeth. Likewise, difficulty inhibiting prepotent behaviors may manifest as maladaptive coping behaviors or violations of social norms. Yet, neither study found a relationship between cognitive flexibility and adaptive behavior [35,59]. These findings begin to demonstrate that certain disrupted EF skills may have downstream effects on adaptive skills necessary for daily living, suggesting interventions targeting EF also may improve adaptive skills. Still more studies are needed in this area.

Importantly, EF abilities were predictive of later functioning as demonstrated by multiple studies. For example, Pierpont and colleagues found that higher working memory performance in school-aged males with FXS predicted greater rate of development of vocabulary and language skills two years later [45]. This highlights that language acquisition in FXS is dependent on the ability to hold verbal representations online as seen in typically developing youths, suggesting the importance of interventions improving working memory in early childhood [86]. Though study authors did not replicate this finding in females with FXS, this may be, in part, due to stronger baseline vocabulary and language skills in females [45]. Additionally, better performance on sustained attention tasks was found to be a strong predictor of lower teacher-rated hyperactivity and problem behaviors and greater prosocial behaviors one year later in school-age males with FXS [50,62]. Likewise, better performance on selective attention tasks in both auditory and visual domains predicted lower parent-reported ADHD symptoms and higher nonverbal IQ 12 months later in school-age males with FXS [64]. This suggests intact early attention skills may be a protective factor against certain comorbid conditions in males with FXS. Interestingly, higher parent-ratings of inattention during preschool years predicted greater improvements in prepotent response inhibition at school-age in males with FXS [14]. Though this finding seems counterintuitive, it suggests the malleability of EF in early development and that weaknesses observed at one timepoint may be related to strengths at another timepoint. It also could suggest that FXS participants with attention issues were identified early and provided treatment, and thus greater improvements were seen across domains. Taken together, findings of relationships between EF and multiple areas of functioning emphasize these skills are likely connected at the brain level and develop very early in childhood. It also highlights the importance of EF in multiple areas of functioning and that early treatment targeting EF may have important, long-lasting impacts. 

#### 3.1.8. Summary of EF Domain Findings 

Few studies directly compared performance between EF domains in FXS, making it difficult to determine whether a consistent profile of EF strengths and weaknesses is evident. This is critical to better understanding whether certain EF deficits emerge as “syndrome specific” to FXS as suggested by some groups [52,87]. Among child males with FXS, verbal working memory was a relative weakness relative to processing speed [33]; however, among adult males with FXS, working memory emerged as a relative strength compared to cognitive flexibility and planning [32]. These studies suggest that working memory may shift from a relative weakness to a relative strength from childhood to adulthood, consistent with findings that the observed gap between verbal working memory performances in FXS and a normative sample narrows during adolescence [33]. A similar observation also was observed in females with FXS with working memory as a relative weakness compared to processing speed in childhood but becoming a relative strength compared to inhibitory control in adulthood [33,38]. This provides important evidence that EF profiles in FXS likely change over the course of development in line with changes in brain maturation and potential compensatory mechanisms, a finding also supported by differences in developmental patterns of specific EF domains in males and females with FXS. For example, Cornish et al. [52] found that for children with FXS, selective and sustained attention increased more with age compared to their inhibition skills, whereas inhibition skills increased more than selective and sustained attention for children with Down syndrome. Additionally, whether developmental and maturation changes also may differentiate males with FXS from females with FXS across certain EF domains also remains unclear and warrants future studies.

Likewise, no study to date has compared strengths/weaknesses profiles across syndromic disorder. Still it is important to note that individuals with FXS demonstrated distractor interference errors during distractor interference and cognitive flexibility measures as well as perseveration errors during distractor interference, selective attention, and cognitive flexibility measures [15,16,18,19,46,48,52,65]. This suggests these EF deficits may reflect underlying cognitive and neural abnormality that manifests itself behaviorally in different contexts of uncertainty/difficulty. Additionally, since perseverative responding was not observed as prominently in other syndromic developmental disorders, it also suggests that perseverative errors may be specific to FXS, reflecting an inability to shift behavior or attention away from a response that has been previously reinforced and/or reflect difficulty managing multiple possible responses [88]. Thus, rather than being separate deficits in ‘inhibitory control’, ‘cognitive flexibility’, and ‘selective attention’, perseverative responding may represent a deficit in a selective component process that impacts multiple EF domains and areas of functioning. As this was one of the most consistent findings across studies, aside from documentation of the presence of EF deficits beyond those expected given cognitive functioning, this warrants further attention in future studies.

### 3.2. Neurobiology of EF Deficits in FXS

Establishing the extent to which deficits on EF measures relate to biologically-based substrates (i.e., FMRP levels, brain anatomy) may provide critical insights into the pathophysiological mechanisms underpinning these cognitive deficits in FXS. Following a review of these findings from previous studies, we will briefly address studies using translational models to assess for EF deficits in *FMR1* Knockout (KO) rodents and then finally summarize potential pathways from gene to behavior.

#### 3.2.1. FMRP

Several studies indicated lower peripheral FMRP expression was associated with more severe EF deficits in both males and females with FXS [39,40,49,51], suggesting a progressive FMRP deficit has a dose-dependent relationship with EF skills. For example, FMRP expression contributed up to 60% variance in EF performance in these studies, suggesting protein levels largely contributed to EF dysfunction, though other neurobiological and environmental factors influence these deficits as well [40]. On the other hand, one study reported cognitive flexibility performance no longer related to FMRP expression once FSIQ was controlled for in a co-ed sample [23]. Given the well-documented relationship between FMRP levels and intellectual ability [8,9,10,11], it suggests general cognitive functioning may have a stronger relationship with FMRP than specific EF skills, like cognitive flexibility. This finding also may be due, in part, to less variation in FMRP levels in males with FXS despite a wider spectrum of cognitive abilities. Thus, the link between FMRP levels and EF deficits may be more evident in females only. Several studies of females with FXS reported that reduced FMRP expression was associated with worse processing speed [39,40] and cognitive flexibility deficits related to lower X activation ratio [34], which directly affects the amount of protein produced. In contrast, relationships between CGG repeat count and EF measures largely were absent [34,47,83], consistent with other studies documenting CGG repeat count is a less reliable biological correlate than FMRP levels. Still, FMRP levels themselves remain limited as taken from blood samples, and thus are a less objective quantification of levels in the brain. Together, these findings provided an important link between causal pathology and observed phenotype of EF deficits in individuals with FXS. 

#### 3.2.2. Structural Brain Imagining Studies

Structural MRI studies consistently have reported increased volume of the caudate nucleus in individuals with FXS [89,90,91,92,93,94,95,96,97,98,99]. The caudate is critical for goal-directed actions, whereby individuals can successfully execute correct behavioral responses and appropriate subgoals [100]. Of note, abnormal caudate volume has been associated with perseverative responding in a number of psychiatric and neurological conditions (for examples, see [101,102,103]), suggesting its role in this behavior in FXS. Caudate volumetric increases appear to occur early in development and persist throughout the lifespan in FXS [96], consistent with findings documenting EF deficits, including perseverative responding, are found from early childhood to adulthood in individuals with FXS. Furthermore, caudate enlargement also has been found to be related to reduced FMRP expression, suggesting a possible progressive dose-dependent relationship between protein and brain volume [92,95]. In conjunction with similar findings of a relationship between reduced FMRP expression and more severe EF deficits, this indicates a potential pathway from reduced FMRP leading to increased caudate to EF dysfunction. 

Individuals with FXS also have demonstrated reduced volume of the cerebellar vermis [92,96,98,104,105], a region with known involvement in working memory, cognitive flexibility, and planning [106]. On the other hand, inconsistent findings have emerged regarding cerebral volume in FXS, though regional differences largely accounted for these inconsistencies [41,91,96,99]. For example, several groups have found increased volume of parietal lobes [92,98] but reduced volume of frontal lobes [94,98,99]. Frontal lobe involvement in EF has been widely-documented (for review see [107]), thus it is not surprising FXS patients have demonstrated reduced volume in this region. The alterations in the parietal cortex previously have been associated with failures to flexibly shift behavior in ASD [108,109], suggesting it may contribute to these deficits in individuals with FXS.

Additionally, previous findings have found altered white matter tract circuitry in individuals with FXS using diffusion tensor imaging [110]. The dorsal–prefrontal circuitry, which includes the caudate, has prominent roles in working memory, set-shifting, and processing speed (for review see [111]). Haas and colleagues [112] also reported greater relative fiber density in the left ventral frontostriatal pathway in young male FXS participants compared to both typically-developing and developmentally-delayed controls. As these findings were observed as early as one year, this suggests frontostriatal white matter tract abnormalities, like increased caudate volume, appear early in life and thus likely reflect alterations in pre- or perinatal brain development. Together, this suggests abnormal development of the frontal-striatum-parietal-cerebellum networks likely is involved in executive dysfunction in FXS [26,113]. 

In a previous study, regional differences in caudate volume related to distinct behavioral phenotypes. For example, the ventromedial caudate of the orbitofrontal cortex (OFC) was associated with social abnormalities, whereas the dorsolateral caudate of the dorsolateral prefrontal cortex (DLPFC) was associated with repetitive speech and behavior, aspects of impaired cognitive flexibility [89]. Though behavioral findings have implicated extensive overlap in neural structures responsible for EF, this finding suggests subtle regional difference in neuroanatomical abnormalities may selectively disrupt processes involved in certain areas of EF. It also may suggest that observed behavioral deficits may arise from numerous neural abnormalities. For example, the OFC also is involved in reward processing [114], which is important in dimensional sorting tasks of cognitive flexibility. Thus, high rates of perseverative errors found in FXS may be maintained by both disruptions to reward processing in OFC and propensity towards repetitive behavior in DLPFC. Still, more studies directly examining the relationships between structural brain abnormalities and clinical phenotypes are needed.

#### 3.2.3. Functional Brain Imaging Studies

Only a few functional brain imaging studies during EF measures have been completed in FXS [26,49,54,60]. Though these studies differed in sample sex, control group, and EF domain, findings consistently demonstrated that FXS patients show a pattern of reduced activation in frontostriatal regions critical for EF. For example, during an n-back task of nonverbal working memory, females with FXS did not exhibit expected increases in frontal activation when cognitive load increased, and this reduced activation was related to worse working memory performance as well as reduced FMRP expression [49]. A similar finding was observed in females with FXS during an inhibitory control task, such that reduced FMRP expression related to both worse inhibitory control performance and greater reductions in prefrontal cortical, basal ganglion, and hippocampal activation. Together, these findings implicate the involvement of FMRP in the disruption of frontal-striatum-parietal-cerebellum circuitry, and in turn, executive dysfunction. As this appears to be a progressive deficit with reduced FMRP expression, the circuitry may be more disrupted and less able to appropriately modulate activity when cognitive load increases. This assertion is further supported by several previous behavioral studies discussed earlier, in which EF performance deteriorated as cognitive load increased (i.e., working memory, selective attention).

Interestingly, several fMRI studies documented evidence of compensatory brain mechanisms to support EF performance in males and females with FXS [26,54,60]. For example, Hoeft and colleagues [54] found that during a go/no-go task of prepotent response inhibition, males with FXS showed reduced right frontostriatal activation, but increased left ventrolateral prefrontal cortex activation compared to both IQ-matched DD and CA controls. These findings occurred in the absence of differences in behavioral performance, suggesting compensatory bilateral activation of prefrontal regions may have improved abilities to withhold prepotent responses in males with FXS. The authors also found that males with FXS who demonstrated greater activation in left ventrolateral prefrontal cortex also had higher levels of FMRP expression. This provides further evidence of the effect of progressive FMRP deficit on EF, suggesting the ability of the brain to develop and use compensatory mechanisms only may be afforded to males with FXS with some FMRP production. Consistent with these findings, previous fMRI studies also have documented that females with FXS with higher levels of FMRP demonstrated increased compensatory activation with recruitment of bilateral (versus left-lateralized) prefrontal regions during a distractor interference task [59]. Females with FXS demonstrated comparable errors to CA females, but reduced reaction times, suggesting they also may have adopted a behavioral strategy to sacrifice speed for sake of accuracy. Though differences in activation patterns for FXS and CA participants also may have been due, in part, to differences in behavioral performance. Thus, several studies provide evidence that some individuals with FXS show compensatory bilateral activation of regions known to support specific EF domains (i.e., prepotent response inhibition, distractor interference, nonverbal working memory) that tracks not only with better behavioral performance, but also with FMRP expression. Taken together, previous structural and functional imaging studies provide critical insight into disrupted brain regions and circuitry that likely contribute to EF deficits in FXS, though the specificity of these findings to FXS and selectivity to distinct brain alterations and corresponding EF deficits remains unclear. 

#### 3.2.4. Potential Mechanisms Underlying EF Deficits in FXS

Together, this section has highlighted the potential critical links between FMRP, brain function, and EF deficits in FXS. FMRP is required for normal dendritic pruning, and its absence can lead to immature synapses, aplastic, and non-specific connections, and presumed aberrant activity within affected structures [7,115,116]. The absence of FMRP is presumed to alter structural integrity of neurons and lead to downstream aberrant neural connectivity [117]. This provides one potential mechanism linking the *FMR1* gene to executive dysfunction through disrupted development of frontal-striatum-parietal-cerebellum circuitry. Still the precise processes underlying this developmental cascade remains poorly understood. FMRP is expressed throughout the cerebral cortex, cerebellum, hippocampus, and thalamus during embryonic development [118,119], suggesting its absence could have widespread neural effects as demonstrated in behavioral findings. Regional differences in volumetric findings may provide key insights into possible divergent trajectories in neurodevelopment. For example, volumetric enlargements may indicate reduced post-natal synaptic pruning, as suggested by findings of increased volume of caudate nucleus and of increased frontostriatal white matter tract density as early as 1–3 years in males with FXS [112]. In contrast, volumetric reductions may indicate pre-natal effects of deficient FMRP leading to disrupted post-natal maturation. 

Additionally, imbalance of excitatory: inhibitory neural activity, including within PFC, repeatedly has been documented in slice, rodent, and human models, yet its relation to cognitive deficits has been sparsely investigated [120,121,122]. Enhanced gamma frequency activity in local circuits during rest and disrupted evoked gamma oscillations have emerged as a relatively conserved and stable biomarkers of neural hyper-excitability in translational models of FXS [123,124]. Gamma oscillations are generated by local synaptic interactions of excitatory and inhibitory neurons and controlled by rhythmic firing of inhibitory interneurons, including parvalbumin positive (PV+) fast-spiking interneurons [125]. Thus, observed neurophysiological alterations in FXS humans and *FMR1* KO mice may reflect failures of PV+ neurons to mediate gamma oscillations. Phasic variation in gamma power in association cortex is known to regulate crucial cognitive functions in mice and humans, and thus alterations in gamma band neurophysiology we observe may contribute to cognitive deficits, including executive dysfunction, in FXS [126,127]. For example, high background gamma in FXS may restrict the neural system’s ability to send high gain signals to alert a change in behavior is needed. Studies of individuals with schizophrenia and mouse models of this disorder have documented the association between PV+ interneuron dysfunction and altered gamma oscillations and EF deficits, including those in working memory and cognitive flexibility [125,127,128,129,130,131]. This provides evidence of failure to phasically increase gamma oscillations needed to perform EF functions due to saturated background gamma activity. Still future translational studies are needed determine if or how well-documented EEG abnormalities of both local and long-distance connections are related to EF deficits in individuals with FXS as well as *FMR1* KO mouse models, as this is critical for drug discovery and novel treatment development.

## 4. Crucial Considerations

The >50 studies of EF in FXS reviewed were completed across a wide range of IQ levels (male: 30–82; female: 46–112) and ages (1–75 years), included both single and co-ed sex samples, and used many different EF measures. Though important findings have emerged with new insights in potential component processes underlying EF deficits in FXS, inconsistences across studies still limit our ability to interpret these findings. Multiple crucial methodological issues and confounding factors that could have affected EF performance in individuals with FXS are addressed, with suggestions for future work in this area.

### 4.1. FXS Sample Characteristics

The inclusion of a wide spectrum of ability levels and ages in EF studies of FXS helps capture the breadth of behavioral and cognitive presentations in this patient population, but also likely confounded findings by potentially washing out effects within specific subgroups of individuals. Factors such as medication usage and co-occurring diagnoses also likely confounded EF performance in individuals with FXS. For example, stimulants may have improved certain aspects of EF like attention, processing speed, and inhibitory control, whereas atypical antipsychotics and benzodiazepines may have punitively impacted aspects of EF like processing speed, attention, working memory, and inhibitory control as previously shown [132]. Yet, the majority of studies did not provide data on medication usage for FXS participants (or control groups), and among those studies that did, medication classes were not specified. This makes interpreting findings challenging, especially as some studies showed on-medication participants performed better than those off-medication [56], while others found the opposite trend [31]. Only three studies excluded for psychotropic medication, which is reasonable given known effects on EF [16,41,50]; however, medication-naïve studies are neither representative or feasible in the FXS population. Thus, it is critical for future studies to address potential confounds of medication usage on performance as well as specifically examine performance by medication class when possible within FXS participants. 

Furthermore, few studies reported or accounted for co-occurring conditions in FXS participants. Because co-occurring conditions like ASD and ADHD likely arise from the FMRP deficit, it is difficult to determine the extent to which the pathophysiological processes underlying EF deficits overlap or differ from those underlying these neurodevelopment disorders, which have their own well-documented EF deficits (for review see [133,134]). It also is possible that affected component cognitive processes clinically manifest as both EF deficits and behavioral presentations of these co-occurring conditions or that the observed EF deficits may reflect cognitive traits of other genetic liabilities superimposed upon the FXS phenotype [135,136]. Though the mechanisms remain unknown, it is not surprising that numerous studies indicated EF deficits worsened with more severe ASD symptoms in individuals with FXS [31,35,36,63,64]. Two previous studies excluded participants with DSM-IV diagnoses and one specifically excluded for ASD [32,46,61], though the majority of previous studies did not take ASD of ADHD symptomatology into account (i.e., using clinical variable as covariate) when assessing for EF deficits. Thus, this latter approach as well as comparing EF performance in FXS participants with and without ASD (or ADHD) may be important considerations for future studies. 

### 4.2. Control Group Selection

In addition to highly variable patient groups, previous studies also were highly variable in their choice of comparison control groups. Using a CA control group was not common (*n* = 12) among studies, as it simply compares groups that by definition operate at different developmental levels. Using a CA control group still may be appropriate in initial studies characterizing how EF in FXS differs from typical development, as done in the majority of structural and functional brain imaging studies in FXS [26,49,60]. In contrast, using an MA control group was the most common approach in previous studies (*n* = 21). Yet, using a MA control group is based on the assumption that acquisition of skills and performance on target variables should be similar between groups despite not matching on CA [137]. However, previous studies demonstrated that developmental profiles of EF often differ between individuals with FXS and MA controls [31], contradicting the assumption. This is especially problematic in a case when a 30-year old FXS participant with a mental age of five years is matched to a six-year old control participant with a similar mental age. Due to possible confounds of maturation and history effects, it would be difficult to determine, for example, whether the absence of a deficit was due to compensatory processes developed over time in the individual with FXS [26,49,60] or whether the presence of a deficit only appeared at certain chronological ages as implicated by some prior findings [31,50]. Another assumption put into question is whether overall MA is representative of current functioning, as van der Molen and colleagues showed that EF performance varied based on whether verbal or nonverbal MA was used in comparison [32]. This suggests previous studies using combined MA comparison group may have over- or under-estimated EF deficits in FXS. Taken together, CA and MA control groups each have their own pitfalls, many of which are difficult to avoid. Matching on both mental and chronological age is the ideal option; however, is not always feasible from a recruitment standpoint, and it is often unclear which types of comparison disabilities should be utilized (e.g., Down syndrome, iDD, etc.).

Additionally, similar issues also arose in studies using iDD or syndromic control groups. Studies widely varied on whether these groups were matched (if at all) on chronological age, mental age, or IQ. The critical problem here is that it assumes differences on target variables are genuine difference between syndromic groups rather than confounds such as differences in developmental trajectory or brain maturation rate. Though using iDD or syndromic control groups carry many advantages, including determining the specificity of findings, additional caution should be made when interpreting findings in future studies that do not control for additional aspects. Overall, choosing appropriate control groups is extremely challenging in FXS studies as usually the most ideal group often is not feasible. Careful consideration and acknowledgment of potential confounds related to control groups is recommended in future studies as these decisions may limit implications of findings. 

### 4.3. Measures 

The majority of measures chosen to assess EF in FXS were either part of a common neurophysiological battery (e.g., Woodcock Johnson III, Wechsler tests) or adapted from these more traditional measures (e.g., day/night task adaptation of Stroop). Many studies also chose commonly used measures that do not have standardized versions (e.g., n-back, antisaccade, go/no-go), and less frequently, studies created new experimental measures [62,64]. Independent of domain or type of measure used, individuals with FXS had reduced completion rates compared to CA and MA controls, especially among males with FXS. However, a smaller percentage of FXS participants completed verbal and nonverbal working memory measures compared to measures of inhibitory control [14,16,22,26,31,34,35,52,53,56,57,59], attention [15,18,19,50,52,56,63], and processing speed [22,31,35,56]. Working memory completion rates were highly dependent on task complexity, with higher load tasks with lower completions rates than lower load tasks [22,31,32], consistent with findings demonstrating worse performance as complexity increased. A similar finding was observed in studies using selective attention measures [56]. Cognitive flexibility and planning measures had among the lowest completion rates (e.g., <30%; [22,23,31,46,48,55]). Of note, individuals who did not complete measures were more likely to have lower MA, higher autistic symptomology, and not taking psychotropic medication [14,22,23,35,63]. This suggests previous studies only captured EF performance in a smaller subset, and perhaps less representative sample, of individuals with FXS. Thus, given the high rate of failures across EF measures, this suggests development of more appropriate measures is needed for individuals with FXS.

Traditional neuropsychological measures have many benefits, including verified psychometric properties, published normative data, and standardization of administration and scoring procedures. Still the vast majority of these measures are not be suitable for the FXS population due to heightened floor effects and task complexity as well as lack of normative data for developmental delay populations [138,139]. In fact, completion rates were lowest among standardized measures compared to adapted or experimental measures. Additionally, many traditional neuropsychological measures assessed multiple domains of executive function simultaneously, making it difficult to determine component processes impaired. For example, the Wisconsin Card Sorting Task (and other dimensional sorting tasks) is primarily a measure of cognitive flexibility; however, working memory is necessary to keep the current rule online, inhibition of distractors and prepotent responding is required to limit perseverative and non-perseverative responses, and both selective and sustained attention are important in selecting responses and staying on task, respectively. Thus, poor performance on the task may be less specific to cognitive flexibility deficits in FXS, but may be due to, in part, other aspects of executive dysfunction. Lastly, many traditional measures often heavily depend on verbal instructions and sometimes even verbal responses, which increases potential confounding factors in this disorder with prominent expressive and receptive language deficits. Overall, the psychometric advantages of traditional neuropsychological measures may not outweigh the challenges associated with using these measures in FXS participants. Thus, careful consideration should be made prior to choosing standardized measures, especially in term of floor effects and specificity of findings. 

Several studies examined feasibility of using standardized computer or application-based testing batteries of executive function abilities, including NIH Toolbox, Cogmed Working Memory, and Kiddie Test of Attentional Performance (KiTAP) [35,56,140]. These electronic batteries had distinct advantages over more traditional neuropsychological batteries, including increased participant familiarity with computer/tablet interface, flexibility in testing positions, button or touch response, limited verbal demands, and increased motivation based on ‘game’ environment. Though developmental extensions for two of the NIH Toolbox measures (e.g., dimensional change card test and flanker) were available for FXS participants and allowed for higher completion rates and lower basals, psychometric properties of the development extensions have not yet been established. This suggests the potential benefit of modifying traditional measures to be more developmentally appropriate for this population, but more studies are needed to confirm the validity of these measures. Though many of these measures did not have such modifications, there still is promise in using batteries based on initial feasibility studies based on findings of high test-retest reliability, convergent and divergent validity, and acceptability among participants [35,56,140], especially when developmental modifications are available. 

Less often, groups adapted standardized or non-standardized versions of EF measures to be more child-friendly and appropriate for use in FXS participants or even rarer, developed their own experimental measures [20,50,53,62,64,65,83]. Among these previous studies, common modifications were implemented, including incorporating a simple story to increase engagement, using visually appealing stimuli, and rewarding correct responses. Given the high levels of completion and reduced floor effects among these studies, it suggests minor modifications may allow for the assessment of EF in a wider range of FXS participants, consistent with findings from the modified versions of NIH Toolbox measures. Though, test-retest reliability and other psychometric properties largely have not yet been established for these performance-based measures, which is a critical aspect to measure selection, especially within clinical trials. Taken together, modified measures appear to be the most appropriate when examining EF in FXS, though the psychometric properties and sensitivity to change over time longitudinally or in response to intervention warrant future study. Importantly, this suggests that the majority of measures used in previous studies are not ideal for individuals with FXS and additional work is needed to develop more appropriate measures of EF in this and similar patient populations.

### 4.4. Scoring and Analysis Method

One reason the majority of measures were not appropriate for individuals with FXS was because adequate scores often could not be obtained from FXS participants due to floor effects. Though this may be an effect of measure, it also suggests alternative scoring or analysis methods are needed when assessing EF in this developmental disorder. Floor effects are well-documented in this population [138], which become particularly problematic when trying to track change over time or in response to treatment, as a large range of low raw scores equate to the lowest standard score. Indeed, many studies instead used raw scores, which has been recommended from several groups when assessing cognitive functioning in this population [141]. Additionally, a promising method was developed to calculate deviation scores based on raw scores in order to better capture cognitive performance in FXS by lowering the floor of the Stanford-Binet fifth edition (SB-5) and Wechsler Intelligence Scale for Children-Third Edition (WISC-III) [138,139]. For example, by expanding the floor of the SB-5, individuals with FXS were noted to have significantly lower verbal working memory performance than was indicated by standard scores, and it became a clear weakness as evidenced by being approximately six standard deviations below the mean, relative to most other domains [138]. Yet, this deviation score approach only has been applied to SB-5 and WISC-III thus far, warranting exploration of its use for other neuropsychological measures of EF, especially as this may be an alternative solution to developing new or modified measures.

In addition, choice of dependent variable is an important consideration when assessing EF in FXS. The majority of studies used one or two variables (e.g., reaction times and correct response rates) to quantify performance, which could greatly simplify the complex processes assessed during EF measures. As a result, relevant factors could be overlooked and thus impede our understanding of mechanisms underlying impairments. For example, categorizing error types proved useful in multiple studies as it showed individuals with FXS had a propensity towards repetitive, or perseverative responding, during distractor interference, cognitive flexibility, and selective attention measures that was not observed in other syndromic disorders like DS and WS [18,19,46,52]. Thus, consideration of additional relevant variables that may better reflect component processes may be important for future EF studies in FXS. Additionally, choice of dependent variable (and measure) is critical to consider in the context of clinical trials in its ability to detect real change when it occurs amid other factors leading to variability/improvement. Relatedly, it also is important to consider whether certain measures and dependent variables reflect more meaningful clinically significant changes as opposed to statistically significant changes. However, our review of the literature demonstrates we remain limited in this regard and future studies helping to determine these answers are critically needed.

### 4.5. Lack of Analogous Paradigms in Translational Studies of Rodent Models of FXS

In order to better understand the mechanistic bridge from gene to behavior in FXS, it is important to examine EF performance in *FMR1* KO mouse models of FXS during translational behavioral measures. The development of clinically- and biologically-relevant behavioral assays comparable to those used in humans is an area that warrants further consideration. Though tests of anxiety, seizure susceptibility, sensorimotor gating, sociability, and sensory hypersensitivity have been readily implemented in FXS rodent models, few have explored executive dysfunction [126]. Moreover, no study to date has examined EF performance in both species using parallel measures. Previous studies have documented EF deficits in *FMR1* KO mice, though findings are more variable than those found in human studies. Additionally, measures used in rodent students often are not analogous to those used in human studies. For example, several studies have reported mild to absent working memory and cognitive flexibility deficits in FXS rodent models [142,143,144] despite the consistency of these findings in FXS humans. One possible explanation for inconsistent findings across species is the use of the Morris water maze to assess nonverbal (spatial) working memory in *FMR1* KO mice [142,145], for which there is no human equivalent. In addition, absence of findings in mice may have been due, in part, to relatively intact nonverbal working memory in FXS humans compared to verbal working memory, especially when cognitive load is low, suggesting Morris water maze may be too easy for the mice to complete.

On the other hand, during a five-choice serial reaction time task [146], *FMR1* KO mice show quicker response times, more false alarms, and more perseverative responding compared to WT mice during reversal trials [147,148]. Yet, perseverative responding normalized with successive training in *FMR1* KO mice, suggesting behavioral intervention similarly may help reduce perseverative behavior in individuals with FXS. Krueger and colleagues [149] also reported increased cognitive flexibility errors during a spatial discrimination reversal learning task in *FMR1* KO mice (though error type was not specified). Thus, difficulty extinguishing a previously rewarded stimulus is consistent with findings from FXS participants during measures of distractor interference, cognitive flexibility, and selective attention, and suggests perseverative responding may be relatively conserved across species. 

Additionally, Krueger and colleagues [149] reported that cognitive flexibility errors were associated with decreased synaptic markers in orbitofrontal and medial prefrontal cortices, and that reductions in postsynaptic proteins preceded expression of cognitive flexibility impairments. These findings suggest a potential causal link between loss of FMRP expression in the PFC and cognitive dysfunction that has only previously been implicated in human imaging studies. Given evidence of the selectivity of specific cognitive flexibility errors in FXS as well as potential insights into gene to behavior pathways, development of parallel measures in individuals with FXS and mouse models may be particularly important. For example, analogous reversal learning paradigms have been used in individuals with ASD and BTBR mouse models of ASD and have identified similar cognitive flexibility deficits in both species [108,149,150]. Currently, our group is piloting the same measure in individuals with FXS, with preliminary findings suggesting increased perseverative errors compared to CA controls (unpublished). This ongoing work in collaboration with groups studying *FMR1* KO mice may be critical to the development of translational biomarkers in FXS. Though examining EF deficits in *FMR1* KO mice is still in its infancy, previous studies offer promising findings that suggest the importance of this area in future research. 

## 5. Conclusions and Future Directions

Previous studies have begun to characterize EF deficits in FXS as well as provide evidence linking FMRP expression to frontal-striatum-parietal-cerebellum circuitry and, ultimately, executive dysfunction. Our review also highlighted that perseverative responding emerged as one the most consistent findings across measures and most specific to FXS. Yet, critical gaps in our mechanistic understanding of these deficits remain. A focused effort on developing translational measures that can be used across species and methods (i.e., behavioral, EEG), is selective towards one EF domain or cognitive process, and minimizes undue burden on the FXS participant is critical to bridging this gap. By advancing our understanding of the pathophysiological processes underlying EF deficits in FXS, as a field we will be better-suited to target EF in treatment studies. Outcome measurement of EF in FXS clinical trials remains in its infancy. Though some traditional neuropsychological measures have demonstrated high test-retest or reproducibility in this patient population (for complete list of measures see [141]), individuals with FXS showed improvement only one measure (i.e., RBANS List Learning) following open label treatment trial with lithium [151]. Still authors even reported that the cognitive battery was too difficult for most FXS participants to complete, suggesting the measures chosen were not appropriate for FXS participants, especially in the context of clinical trials. A recent review of outcome measures suggested potential outcomes measures in FXS treatment studies [152], including specific KiTAP and Woodcock–Johnson subtests. However, these measures only have been used in FXS during initial feasibility studies [56], suggesting studies are needed to confirm their appropriateness in this patient population, especially during treatment trials. Thus, as a field it is important to critically examine the state of literature, and focus future work on identifying (or developing) measures of EF with high test-retest reliability and construct validity that also link to hypothesized neurobiological mechanisms as this would allow for greater potential of success in future clinical trials.

## Figures and Tables

**Table 1:** Executive function measures and findings in individuals with FXS.

Executive Function Domain	Measure ^1^	Findings ^2^		References
**Working Memory**		**Males**	**Females**	
Verbal	WJ-III Memory for Words; NIH Tool Box List Sorting; RAKIT Learning Names; WMTB Nonword Repetition; RBMT Story Recall; WMS-R Logical Memory I; SB-IV Sentence Memory; Weschler Digit Forward, Digit Backward; WJ-III Auditory Memory; WISC Letter-Number Sequencing; KABC Verbal Number Recall; WISC, WAIS Arithmetic; TEA-Ch Elevator Counting	vs. CA ↓ vs. MA ↓ vs. iDD ↓ vs. DS ↓≈	vs. CA ↓ vs. IQ ≈ vs. Enviro ≈	[15,16,21,22,31,32,33,34,35,36,37,38,39,40,41,42,43,44,45,46]
Nonverbal/Visuospatial	Leiter Spatial Memory; CANTAB Spatial Span; KABC Spatial Span; SB5 Spatial Span; Delayed-no-matching-to-position; Toy Recall	vs. CA ↓ vs. MA ↓ vs. iDD ↓ vs. DS ↓≈	vs. CA ↓	[16,21,22,31,32,34,41,42,47,48,49]
Nonverbal/Visuoperceptual	Delayed-non-matching-to-sample; WMS-R Figural Memory I; Fish color; Sequence Memory Task; n-back; SB-IV Bead Memory; Card Task; Hidden Object Memory Test	vs. CA ↓ vs. MA ↓ vs. DS ↓≈	vs. MA ↓ vs. Enviro ↓≈	[15,16,34,35,37,43,49,50,51]
**Inhibitory Control**				
Prepotent Response Inhibition	KiTAP Go/No-go; Lab-TAB Snack Delay; Antisaccade; TEA-Ch Walk task	vs. CA ↓ vs. MA ↓ vs. iDD ≈ vs. DS ↓	vs. CA≈	[14,16,26,52,53,54,55,56]
Distractor Interference	Stroop, Day/Night Task, CNT; Visual Selection; TEA-Ch Same-Opposite task; KiTap Distractibility, NIH Toolbox Flanker	vs. CA ≈↓ vs. MA ↓ vs. iDD ↓ vs. DS ↓	vs. IQ ↓≈ vs. Enviro ↓ vs. TS ≈	[16,22,31,34,35,52,55,56,57,58,59,60]
Cognitive Flexibility	NIH Toolbox Dimensional Change Card Sort Test; CANTAB IED; Wisconsin Card Sorting; iTap FlexiContingency Naming Task Subtest 3; Kbility	vs. CA ↓ vs. MA ↓ vs. iDD vs. DS ↓≈ vs. PWS ≈ vs. Enviro ≈	vs. Enviro ↓≈	[15,22,23,31,32,34,35,46,48,55,56,61]
**Attention**				
Sustained	CPT; KiTap Vigilance; KiTAP Sustained Attention; WIAT Vigilance; WATT Vigilan task; Elevator Counting	vs. CA ↓ vs. MA ↓ vs. DS ↓≈	n/a	[15,16,37,50,52,56,62,63,64]
Selective	Symbol Search, Cancelation, Map Search, WATT Visearch task; WISC Symbol Search; KiTAP Visual Scanning; TEA-ch Map Search	vs. CA ↓ vs. MA ≈ vs. DS ↑ vs. WS ↑	n/a	[15,16,18,19,52,56,65]
Divided	KiTAP Divided Attention; WATT Visearch dual task	vs. MA ↓ vs. DS ≈	n/a	[16,56]
Planning	NEPSY Tower; WJ-III Planning; CANTAB Stockings of Cambridge	vs. MA ↓	n/a	[22,31,32]
Processing Speed	WISC Coding, Cancelation; NIH Toolbox Pattern Comparison; Reaction Time from CNT; KiTAP Alertness	vs. CA ↓ vs. MA ≈	vs. CA ↓	[22,31,33,35,56]

^1^ Abbreviations for cognitive measure used: CANTAB—Cambridge Neuropsychological Test Automated Battery [66], CNT—Contingency Naming Task [67], CPT—Continuous Performance Test [68], IED—Intra-/Extra-Dimensional task, KABC—Kaufman Test of Attention Performance for Children [69], Lab-TAB—Laboratory Temperament Assessment Battery [70], NEPSY—Developmental Neuropsychological Assessment, RAKIT—Revised Amsterdamse Kinder Intelligence Test, RMBT—Rivermead Behavioral Memory Test [71,72], SB-IV—Stanford-Binet Intelligence Scale-4th Edition [73], SB-5—Stanford Binet Intelligence Scale-5th Edition, TEA-Ch—Test of Everyday Attention for Children [74], WAIS—Wechsler Adult Intelligence Scale, WATT—Wilding Attention Tasks [75], WIAT—Wechsler Individual Achievement Test, WISC—Wechsler Intelligence Scale for Children [76], WJ-III—Woodcock Johnson [77], WMS-R—Wechsler Memory Scale-Revised [78], WMTB—Working Memory Test Battery, Third Edition [79]; ^2^ ↓—indicates participants with FXS performed worse than specified control group, ↑—indicates participants with FXS performed better than specified control group, ≈—indicates participants with FXS performed similar to specified control group, ≈↓—indicates mixed findings; Participant type: CA—chronologically-aged match controls, MA—mentally-aged matched control, iDD—control with idiopathic developmental delay, DS—control with Down Syndrome, WS—control with William Syndrome control, PWS—control with Prader–Willi Syndrome, Enviro—environmental control (unaffected in-house relative).
